# Development of decision aids for female *BRCA1* and *BRCA2* mutation carriers in Germany to support preference-sensitive decision-making

**DOI:** 10.1186/s12911-021-01528-4

**Published:** 2021-06-05

**Authors:** Sibylle Kautz-Freimuth, Marcus Redaèlli, Kerstin Rhiem, Andrea Vodermaier, Lisa Krassuski, Kathrin Nicolai, Miriam Schnepper, Violetta Kuboth, Julia Dick, Vera Vennedey, Regina Wiedemann, Rita Schmutzler, Stephanie Stock

**Affiliations:** 1 Institute for Health Economics and Clinical Epidemiology, The University Hospital of Cologne, Gleueler Straße 176-178, 50935 Cologne, Germany; 2 Centre for Familial Breast and Ovarian Cancer, Centre for Integrated Oncology (CIO), Faculty of Medicine, University of Cologne, University Hospital of Cologne, Kerpener Straße 62, 50937 Cologne, Germany; 3 School of Population and Public Health, The University of British Columbia, 2206 East Mall, Vancouver, BC C6T 1Z3 Canada

**Keywords:** *BRCA1*, *BRCA2*, *BRCA1/2* mutation carriers, Decision aids, Development process, Hereditary breast and ovarian cancer, Preference-sensitive decisions, Preventive options

## Abstract

**Background:**

Women with pathogenic *BRCA1* and *BRCA2* mutations possess a high risk of developing breast and ovarian cancer. They face difficult choices when considering preventive options. This study presents the development process of the first decision aids to support this complex decision-making process in the German healthcare system.

**Methods:**

A six-step development process based on the International Patient Decision Aid Standards was used, including a systematic literature review of existing decision aids, a topical medical literature review, preparation of the decision aids, focus group discussions with women with *BRCA1/2* mutations, internal and external reviews by clinical and self-help experts, and user tests. All reviews were followed by iterative revisions.

**Results:**

No existing decision aids were transferable to the German setting. The medical research revealed a need to develop separate decision aids for women with *BRCA1/2* mutations (A) without a history of cancer (previvors) and (B) with a history of unilateral breast cancer (survivors). The focus group discussions confirmed a high level of approval for the decision aids from both target groups. Additionally, previvors requested more information on risk-reducing breast surgery, risk-reducing removal of both ovaries and Fallopian tubes, and psychological aspects; survivors especially wanted more information on breast cancer on the affected side (e.g. biological parameters, treatment, and risk of recurrence).

**Conclusions:**

In a structured process, two target-group-specific DAs for previvors/survivors with *BRCA1/2* mutations were developed to support decision-making on risk-adapted preventive options. These patient-oriented tools offer an important addition to existing specialist medical care in Germany.

**Supplementary Information:**

The online version contains supplementary material available at 10.1186/s12911-021-01528-4.

## Background

Women who carry inherited pathogenic mutations in the *BRCA1* or *BRCA2* genes possess an increased risk of developing breast cancer (BC) and ovarian cancer (OC) compared to women of the general population without these mutations. Up to the age of 80, their average cumulative risk to develop BC is approximately 70% (*BRCA1* or *BRCA2* mutation) and the average lifetime risk to develop OC is approximately 44% (*BRCA1* mutation) or 17% (*BRCA2* mutation) [1]. As a rule of thumb, women with *BRCA1/2* mutations who have no personal history of cancer (previvors) [2] develop BC or OC around 20 years earlier compared to women who develop sporadic BC or OC. For women with *BRCA1/2* mutations who have a personal history of unilateral BC (survivors) [3], the average cumulative risk to develop BC on the healthy side (contralateral BC) within 20 years of initial diagnosis is approximately 40% (*BRCA1* mutation) or 26% (*BRCA2* mutation) [1].

Women who receive a positive genetic test result confirming a pathogenic mutation in a risk gene face difficult and far-reaching decisions [4]. They need to decide which preventive measures to take and when. In the following, the generic term ‘preventive option or measure’ applies to all measures that can be offered to women with *BRCA1/2* mutations either to reduce the risk of breast or ovarian cancer or for breast cancer screening. The preventive options available are an intensified breast cancer screening programme for previvors, an intensified breast cancer screening and aftercare programme for survivors, and risk-reducing surgeries for both groups. Internationally, the use of anti-oestrogenic drugs such as tamoxifen or aromatase inhibitors for primary prevention is also discussed. So far, there is no conclusive evidence of a clear benefit as primary prevention in previvors and recommendations vary internationally [5–9].

Intensified breast cancer screening detects BC at an early, potentially curable stage in almost 85% of cases, but does not reduce the risk of BC [10]. Risk-reducing removal of healthy mammary glands (risk-reducing bilateral mastectomy) significantly reduces the risk of BC and gives women with *BRCA1* mutations a survival benefit [11, 12]; however, it also results in permanent loss of the breast and requires additional decisions regarding operations and breast reconstruction processes. For survivors, risk-reducing removal of the healthy breast (contralateral mastectomy) can reduce the risk of contralateral BC and improve overall survival [13]. However, the process of deciding whether to choose this option is made especially complicated by potential competing risks, such as the risk of BC relapse on the affected breast side. Survivors then face the decision of weighing the risk of relapse on the affected side against the benefits of risk-reducing contralateral mastectomy of the healthy side. As there is no effective screening method for OC [14–17], the only available preventive measure is risk-reducing removal of both ovaries and Fallopian tubes (risk-reducing bilateral salpingo-oophorectomy). This surgical procedure significantly reduces the risk of OC and provides a survival benefit [18], but results in loss of fertility and may induce premature menopause.

Each preventive measure comes with different benefits and risks, which each woman rates differently. The same applies to breast reconstruction following mastectomy, family planning, and steps for handling side effects [4]. As such, women with *BRCA1/2* mutations are faced with preference-sensitive decisions that can lead to decisional conflict, hesitation, dissatisfaction, regret, and assigning blame to therapists [19–23]. A decision is deemed preference-sensitive if the subject has a choice of two or more medical options of nearly equal value that offer no clear advantage in terms of clinical outcome, or that are perceived differently depending on the subject’s own preferences and values [24, 25]. To foster high quality decision-making in such situations, it is important, to provide women with sufficient evidence-based medical information that enables them to get a realistic picture of their risk constellations and their options [25]. On the other hand, it is also important to take into account personal factors such as their individual life situation, family and psychological stressors, as well as their individual values and preferences [19, 25–27].

In order to support decision-making on preventive measures for women with *BRCA1/2* mutations and improve patient information and decision quality, an increasing number of supporting tools are being implemented worldwide; particularly decision aids (DAs). Evidence-based DAs can effectively support decision-making with regard to therapeutic or screening options. This has been demonstrated in a Cochrane Review covering 105 studies with a total of 31,043 participants [28] indicating that DAs improve understanding of the available options and the accuracy of risk assessments. They can also improve decision-related criteria. Decisional conflicts resulting from a feeling of not being sufficiently informed are reduced, as is indecision about patients’ values. Fewer patients remain passive during their decision-making process. A systematic review focusing on the effectiveness of DAs for women with *BRCA1/2* mutations reported that decision-making is primarily supported by improving decision-related effects [29]. In principle, DAs seem suitable for improving health literacy among target groups.

In Germany, around 70,000 women develop BC and around 7400 women develop OC every year [30, 31]. Approximately 30% of these women have a family history of BC and/or OC [32]. In around 24% of these patients, genetic testing will identify a pathogenic mutation in the *BRCA1* or *BRCA2* genes [33]. Healthy women with a strong family history of BC/OC are also offered genetic testing [32]. A clear positive genetic test result allows women both with and without a history of BC, to consider whether, and if so, how to address their increased risk of BC, BC in the healthy breast and OC.

Genetic testing at the German Consortium for Hereditary Breast and Ovarian Cancer’s (GC‐HBOC) centres is embedded in a specialised counselling and care concept that ranges from individual risk prediction to discussion of risk-adapted preventive measures and their respective consequences [8, 9, 14, 34]. The counselling takes the form of a personal doctor/patient consultation. Women are also provided with some written information. However, to date, no structured tools such as DAs are available in Germany to help these women make informed decisions based on their individual values.

The aim of this project is to support the decision-making process for women carrying *BRCA1/2* mutations and to enable them to make quality decisions. To support these women we developed evidence-based DAs that are compatible with the German healthcare context and the German guidelines in a structured process based on the criteria of the International Patient Decision Aid Standards (IPDAS).

## Methods

### Development team

The development was conducted by a multidisciplinary team of experts from healthcare research, medicine, psychology and nursing science, and specialists in obstetrics and gynaecology in the field of hereditary BC and OC. The latter have extensive experience in specialist medical care for the target group and are leading members of the GC-HBOC.

### Development process and task distribution

**Fig. 1:**
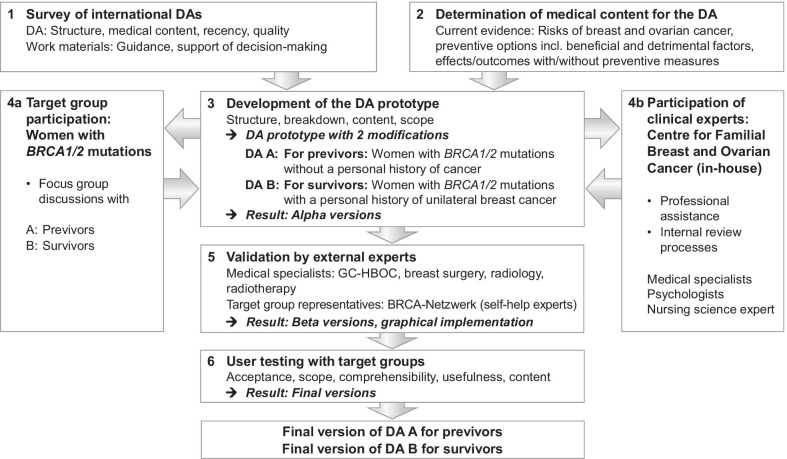
Six-step work process for the development of the DAs for women with *BRCA1* or *BRCA2* mutations. *BRCA1/2* BReast CAncer gene 1 and 2, *DAs* decision aids, *GC-HBOC* German consortium of hereditary breast and ovarian cancer

The development followed a six-step work process (Fig. 1) based on the approach described by the IPDAS Collaboration [35–37]. The Ottawa Decision Support Framework [38, 39] served as the basis for the theoretical framework. The quality requirements were based on the IPDAS criteria and the Ottawa Health Research Institute’s (OHRI) Workbook on Developing and Evaluating Patient Decision Aids [35, 36, 38, 40, 41].

The development team formulated basic requirements for the DA with regard to targets, format, structure, content, and quality and conducted literature reviews (Steps 1, 2), conceptualisation, preparation and revisions of prototypes, alpha, beta and final versions of the DAs (Steps 3, 5, 6), focus group discussions and user tests (Steps 4a, 6). The clinical experts of the development team assisted the entire development process, paying particular attention to the clinicians needs, and performed the internal medical plausibility reviews (Step 4b).

Independent external women with *BRCA1/2* mutations (layperson-patients) were involved in Steps 4a and 6. The external validation (Step 5) used to check that the DAs were medically sound and up-to-date was carried out by independent external medical specialists. Self-help experts from the BRCA-Netzwerk^1^ were brought on board as “professionalised laypersons” (expert patients) with extensive experience in the situations of women with *BRCA1/2* mutations and the basic medical and practical skills required for handling them. None of the persons brought in for the external reviews were involved in the development process.

### Target definitions

Initially, the requirements for the DA content were defined as shown in Table 1.

**Table 1:** Defined requirements for the DA content

Target	Target definition
Target group	The DA should be designed for female previvors and survivors with pathogenic *BRCA1* or *BRCA2* mutations
Target decision	The addressed decision situation should be the choice of an individual preventive strategy
Decision-making-related target	The DA should help previvors and survivors make informed decisions and clarify their individual values and preferences
Information/content-related target	The DA should address the risks of breast cancer and ovarian cancer and present the respective preventive options, including their consequences. Existing data on lifetime and on age and time-related risks should be taken into account in order to facilitate the women’s risk perception within a foreseeable period of time
Needs-related target	The DA should fulfil the specific needs of the target group
Evidence-related target	The medical content should be based on the currently applicable German S3 and S2 guidelines and, where necessary, on additional data with a high level of evidence; preferably data with an expected level of evidence of IIb and higher (e.g. systematic reviews based on RCTs, meta-analyses, RCTs, high quality cohort studies)

*BRCA1* and *BRCA2*: BReast CAncer genes 1 and 2; DA: decision aid; previvors: women with pathogenic *BRCA1* or *BRCA2* mutations without a personal history of cancer; survivors: women with pathogenic *BRCA1* or *BRCA2* mutations with a personal history of unilateral breast cancer; RCTs: randomised controlled trials; S3 guidelines: systematically elaborated evidence-based guidelines for medical recommendations, elaborated with all elements of systematic development (highest quality level); S2 guidelines: systematically elaborated evidence-based guidelines for medical recommendations (S2e)

### Work process

#### Step 1: Survey on existing decision aids

To gather information on international DAs for women with *BRCA1/2* mutations, a systematic literature review was conducted. The aim of the review was to assess the compatibility of the available DAs and their structure and/or content with the current German healthcare system.

Altogether, six databases were searched (MEDLINE, Embase, PsycINFO, CINAHL, ERIC, Cochrane Database of Systematic Reviews). Manual search for relevant DAs was performed on the websites of the Institute for Quality and Efficiency in Health Care (IQWiG, www.iqwig.de) and the OHRI Decision Aid Research Group (www.ohri.ca/decisionaid). The review covered DAs in German and English. There were no restrictions on the date or type of publication.

The review included DAs for women with *BRCA1/2* mutations aged between 18 and 70 and studies on development, structuring, implementation and evaluation of these DAs. The term “decision aid” was defined in accordance with IPDAS [35]. Screening was conducted by two independent reviewers based on the PRISMA statement [42]. Discrepancies were discussed and resolved with a third reviewer.

The identified DAs were assessed based on formal, structural, medical-content, and quality criteria and rated in terms of their suitability as a basis for adapting/developing a DA for the German healthcare system. A search for incorporated decision-supporting worksheets (guidance) that focussed on comparing the preventive options, clarifying personal values, and doctor/patient consultation aids was also conducted [43].

The medical-content assessment was based on procedures and evidence-based recommendations for Germany [8, 9, 14] in coordination with the medical specialists of the development team. The quality assessment was conducted using the IPDAS instrument short form (IPDASi-SF) which consists of 16 criteria [41].

#### Step 2: Determination of the medical content for the decision aids

The medical content was determined in topical literature searches. It was based on the latest available evidence on the risks of developing BC, contralateral BC and OC and on preventive options including benefits and risks with regard to the following outcomes: Incidence reduction, reduction of BC/OC-specific morbidity and mortality, overall mortality, quality of life, and side effects. References for the German health care context were data from the Centre for Cancer Registry Data of the Robert Koch Institute (RKI) [30, 31], German S3 and S2 guidelines [8, 9, 14] and information from the GC-HBOC [34]. In general, the search followed a top-down approach. The German S3 guidelines were used as the basis, as these represent the evidence-based consensus in Germany at the highest quality level of methodological development. Consistencies, discrepancies or additional information of possible relevance were searched for in the S2 guidelines. Data that may not yet have found their way into the available guidelines were searched via MEDLINE and google scholar, with preference given to studies with an expected evidence level of IIb and higher, if available. The resulting medical content was reviewed and approved by the medical experts of the development team for clinical and patient relevance.

#### Step 3: Prototype development

The results from Steps 1 and 2 were used to define the structure, content breakdown, medical and decision-supporting contents, overall scope, and format and draw up a prototype, based on established tools for the development and evaluation of evidence-based patient information [40, 44–47]. With the assistance of the medical specialists of the development team, the medical contents that emerged from the literature search in Step 2 were assessed in terms of clinical and patient relevance in specialist counselling and accepted and converted into information comprehensible to laypersons. Since the medical data obtained in Step 2 suggested that it is reasonable to target previvors and survivors separately due to their different baseline situations and risk constellations, two DA prototypes were designed for each target group.

#### Step 4: Participation of target groups and internal clinical experts

In Step 4a, each of the DA prototypes was discussed in two guideline-based focus group discussions with previvors and survivors respectively, and their attitudes, expectations and experiences were explored openly in relation to the prototypes [48–50]. The aims of this process were to have both DA prototypes discussed and evaluated from a target group-specific retrospective user perspective in terms of their contents and needs. This served to improve and add/remove parts of the prototypes and determine the needs of the specific target groups.

Voluntary participants were recruited by a clinical psychologist of the Centre for Familial Breast and Ovarian Cancer at the University Hospital of Cologne via phone call. The sample was selected using the theoretical guided sampling variant of the purposive sampling approach [51]. The participation requirements were: Clearly pathogenic *BRCA1/2* mutation, experience with the decision-making process for choosing a prevention strategy, no personal history of cancer (for participation in the focus groups discussing DA A), a personal history of BC (for participation in the focus groups discussing DA B), receipt of genetic test result at least 3 months prior to the focus group date to ensure a minimum distance from news of a mutation, emotional stability as rated by the psychologist during the phone call, and written informed consent. One week before the focus group discussions, the participants received the DA prototype to review.

The focus group discussions were audio-taped, transcribed verbatim [52] and evaluated according to Mayring’s qualitative content analysis [53] by two independent assessors. MAXQDA software was used for analysis. A silent observer for each focus group generated a postscript to record situational and non-verbal aspects such as the mood, and the behaviour of the groups and moderators [54].

The results were used to comprehensively revise and add to the DA prototypes before they were presented to the clinical experts of the development team in Step 4b for a professional assessment. Following further revisions, these were used to create the alpha versions in manuscript form.

#### Step 5: Validation by external experts

The alpha versions were checked by independent, external medical specialists in the fields of breast surgery (n = 2), oncological and senological radiology (n = 1), radiotherapy (n = 1), and hereditary BC/OC (n = 2) and self-help experts (n = 3) to ensure that they were medically correct, up to date and patient-oriented. All the review results were discussed and accepted by the development team.

#### Step 6: User testing

The revised alpha versions were used to create graphical beta versions. These were tested by independent, external test readers from the respective target groups to determine their comprehensibility, usefulness, and the acceptance of their contents [55, 56]. The advertisement for volunteer readers was posted by the BRCA-Netzwerk. Each reader received one test copy. Two weeks later, a guideline-based, semi-structured telephone interview was conducted with each reader. The questions covered their general impressions and assessments on length, amount of information, comprehensibility, balance, usefulness, satisfactory nature, and specific content of the DA. The responses were documented in pseudonymised form. The results were used for the final revision. The final DA versions were printed as A5, profile, paper brochures.

Further details on the methodology of Steps 1 to 6 can be found in Additional file 1.

## Results

### Step 1: Survey of existing decision aids

**Fig. 2:**
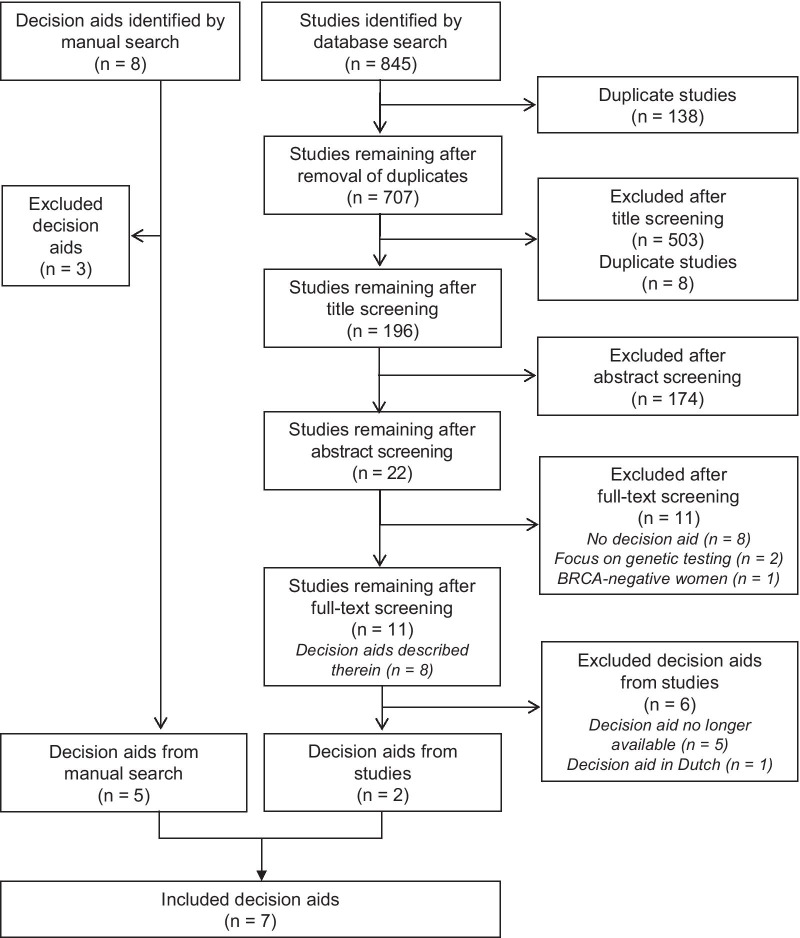
Results of the systematic literature review to identify available DAs for women with *BRCA1/2* mutations based on the PRISMA statement [42]; as of 6 December 2016. DAs: decision aids; Search strategy: (BRCA1 OR BRCA2 OR BRCA1/2 OR BRCA) AND (decision making OR decision aid OR decision support tool OR decision support technique OR decision support techniques)

A total of 845 studies were retrieved (Fig. 2). Following exclusion of duplicates and screening of title, abstract and full text, eleven studies that deal with a DA remained. Two of the DAs described were obtainable through the developer/study author. Eight DAs were identified using manual searches. Five of these were included. Thus, at the time of the review, seven DAs published between 2006 and 2016 were available for further analysis. A list of the identified DAs is provided in Additional file 2.

Table 2 provides an overview of the basic structural elements found in the identified DAs.

**Table 2:** Basic structural elements in the identified DAs

Main topic	Basic structural elements	References (DA)
Target group	Women with *BRCA1/2* mutation	[57, 58]
	Women at high risk of breast cancer	[59, 61, 64]
	Women at high risk of ovarian cancer	[60, 62, 63]
Addressed target decision	Preventive options related to breast and ovarian cancer	[58]
	Preventive options only/mainly related to breast cancer	[57, 59, 61, 64]
	Preventive options only/mainly related to ovarian cancer	[60, 62, 63]
Medical information	Family history of breast and/or ovarian cancer (explanations, even if brief)	[59–64]
	Impact of a pathogenic *BRCA1/2* mutation (reason for high risk of breast and/or ovarian cancer)	[57–64]
	Average morbidity rates for breast and/or ovarian cancer (text and/or number values and/or graphics)	[57, 58, 61–64]
	(a) Lifetime risks	(a) [57, 58, 61–63]
	(b) Time and/or age-related morbidity rates	(b) [62–64] (example information)
	Preventive options (explanations, even if brief)	[57–64]
	Outcomes with and without preventive measure (text and/or number values and/or graphics)	[57, 58, 60–64]
	Consequences of the different preventive measures (text and/or number values and/or graphics)	[57–64]
	Hormone replacement therapy to counteract consequences of risk-reducing removal of both ovaries and Fallopian tubes	[60, 62, 63]
	Benefits and risks of the different preventive measures and/or comparison of the options (option grid)	[57, 59–64]
	Personal stories (testimonies)	[61, 64]
	Appendix with fact boxes	[57, 61]
Tools for decision-making support	General information on dealing with decision-making	[57, 64]
	Step-by-step guide to decision-making	[57, 62, 63]
	Tools to assist with clarifying own values and preferences, e.g To reflect on personal opinions regarding the advantages and disadvantages of the options by answering set response options (box-ticking) and/or using note spaces for free-form reflections To reflect on the current tendency towards or against a certain option	[57, 61–63]
	Question lists addressing medical information and/or doctor-patient dialogue	[60, 64]
Other	Glossary	[57, 58]
	Contact addresses and/or internet links	[58, 62–64]
	References, further literature	[57–64]

*BRCA1/2* BReast CAncer gene 1 and 2, *DA* decision aid; [59–61]: a current version dated 2019 is available

Table 3 summarises the basic medical contents of the identified DAs. None of the DAs met all the predefined requirements and target definitions, and only parts of the German guideline recommendations [8, 9, 14] were reflected. For instance, only two of the DAs explicitly addressed women with *BRCA1/2* mutations [57, 58], only one addressed both BC and OC risks [58]. Two DAs did not specify any risks [59, 60], four mentioned lifetime risks of BC [57, 58, 61] or OC [62, 63], and none of them provided adequate information on age or time-related risks, specifically addressed survivors, discussed the risk of contralateral BC, or explained competing risks. Only one DA included breast ultrasound as a part of the breast cancer screening regimen and information on surgical methods used for risk-reducing mastectomy and breast reconstruction [64]. Four DAs mentioned preventive medication with anti-oestrogenic drugs (such as tamoxifen) as a preventive option for BC in previvors [57, 59, 61, 64], which is not compatible with the German S3 guidelines [8]. Two DAs mentioned screening methods for OC [60, 62, 63], which is not compliant to the German S3 guidelines [14]. Due to the limitations, new DAs were developed that discuss risk-adapted prevention options for both BC and OC and are compatible with the current German guidelines.

**Table 3:** Basic medical contents of the identified DAs

DA: Author/developer, year of publication [reference]	Kurian, 2011 [58]	Metcalfe, 2006 [57]	Hereditary Cancer Clinic et al, 2012^###^ [64]	Healthwise, 2016^#^ [61]	Mayo Clinic, 2016^#^ [59]	Cardiff University, 2014^##^ [62, 63]	Mayo Clinic, 2014^#^ [60]
Format	Online tool for individualisation	Brochure/PDF	Brochure/PDF	Online text	Online text	Online tool for individualisation	Online text
Target group	Women with *BRCA1/2* mutations; differentiation between *BRCA1*and *BRCA2 *mutation Not affected by cancer (previvors)	Women with *BRCA1/2 *mutations; no differentiation between *BRCA1*and *BRCA2 *mutation Not affected by cancer (previvors)	Women with a strong family history of breast cancer and -*BRCA1/2 *mutations -Personal history of unilateral breast cancer -Personal history of multiple breast biopsies -Breast cancer anxiety	Women at high risk for breast cancer with -Family history of breast cancer -*BRCA1/2* mutations	Women at increased risk for breast cancer with -Personal history of unilateral breast cancer -Family history of breast cancer -*BRCA1/2 *mutations -Personal history of LCIS -Personal history of radiotherapy at the age of 10 to 30 -Dense breast tissue	Women at increased risk of ovarian cancer with -*BRCA1*or *BRCA2 *mutations -Being from a Lynch syndrome family -No genetic test -Uninformative genetic test -Negative genetic test	Women at increased risk of ovarian cancer with -*BRCA1 *or *BRCA2 *mutations -Strong family history of breast cancer and ovarian cancer without known genetic alteration -Strong likelihood of gene mutation, but no genetic testing
Addressed cancer risk	Breast cancer risk^a^ Ovarian cancer risk^a^	Breast cancer risk	Breast cancer risk	Breast cancer risk	Breast cancer risk	Ovarian cancer risk^b^	Ovarian cancer risk
Information on cancer risks							
*Lifetime risks of breast cancer*							
No mutation^§^	10–12 of 100	11 of 100	8 of 100	12 of 100	0	0	0
*BRCA1 *mutation	47–85 of 100	0	0	55–65 of 100	0	X^c^	0
*BRCA2 *mutation	40–85 of 100	0	0	45 of 100	0	X^c^	0
*BRCA1/2 *mutation	0	80 of 100	0	40–85 of 100	0	0	0
*Risks of contralateral breast cancer*							
No mutation^§^	0	0	0	0	0	0	0
*BRCA1 *mutation	0	0	0	0	0	0	0
*BRCA2 *mutation	0	0	0	0	0	0	0
*BRCA1/2 *mutation	0	0	0	0	0	0	0
*Lifetime risks of ovarian cancer*							
No mutation^§^	1–2 of 100	0	0	0	0	0	0
*BRCA1 *mutation	39–46 of 100	0	0	0	0	X^c^	0
*BRCA2 *mutation	11–27 of 100	0	0	0	0	X^c^	0
*BRCA1/2 *mutation	0	40 of 100	0	0	0	0	0
*Further risk information*							
Time-related risks	0	0	(X)^d^	0	0	0	0
Age-related risks	0	0	(X)^d^	0	0	(X)^d^	0
Competing risks	0	0	0	0	0	0	0
Information on preventive measures - breast cancer							
*Intensified screening*							
Self-examination^y^	0	X	(X)^e^	0	X	0	0
Medical examination	0	X	(X)^e^	(X)^f^	X	0	0
Breast MRI	X	X	X	X	X	0	X^g^
Mammography	X	X	X	X	X	0	X^g^
Breast ultrasound	0	0	X	0	0	0	0
*Risk-reducing surgery*							
Mastectomy	X^h^	X^h^	X^i^	X^h^	X^i^	0	X^h^
Salpingo-oophorectomy^y^	X^j^	X	0	X	0	X^m^	0
Oophorectomy^y^	X^k^	0	0	X^l^	X	0	X
*Preventive medication*^*y*^							
Tamoxifen	0	X	0	X^n^	X	0	0
Raloxifen	0	(X)^o^	0	X^p^	X^p^	0	0
Aromatase inhibitors	0	(X)^o^	(X)^q^	X^p^	X^p^	0	0
*Other*							
No oral contraception^y^	0	0	X	0	0	X	X
No hormone replacement therapy^y^	0	0	X	0	(X)^r^	X^s^	X^t^
Information on preventive measures -ovarian cancer							
*Screening*^*y*^							
Transvaginal ultrasound	0	0	0	0	0	(X)^u^	(X)^v^
CA125 testing	0	0	0	0	0	(X)^u^	(X)^v^
*Risk-reducing surgery*							
Salpingo-oophorectomy	X^j^	X^w^	0	X	0	X	X
Oophorectomy^y^	X^k^	0	0	X	X	0	0
*Other*							
Oral contraception	0	0	0	0	0	X	X
Hormone replacement therapy^y^	0	0	0	X^x^	0	X	X
Further information							
Breast surgery, breast reconstruction	0	0	X	0	0	0	0
Adnexa surgery	0	0	0	0	0	0	0

*BRCA1/2*: BReast CAncer gene 1 and 2; *DA*: decision aid; *LCIS*: lobular carcinoma in situ;* MRI*: magnetic resonance imaging;* previvors*: women with pathogenic *BRCA1*or *BRCA2* mutations without a personal history of cancer

X: Yes, (X): Yes, but with limitation; 0: No/not applicable

^#^Current version dated 2019 available

^##^Example online version of the DA no longer available

^###^Developers: Hereditary Cancer Clinic, Prince of Wales Hospital, Centre for genetics education, NSW Health, Royal north shore hospital

^§^No mutation: general population

^a^Calculates average rates for mortality and survival after inputting patient characteristics and planned preventive option(s), compares to "no preventive intervention" and "no mutation"

^b^Provides individualized information after inputting personal risk characteristics, age and personal history of breast cancer

^c^States risk rates for individualised situation (applicable mutation, cancer history, woman’s age)

^d^Example information on age and/or time-related risk of disease

^e^States on p. 6: "regular breast examinations" (self/medical examination not specified)

^f^"checkups 1 to 2 times a year", not specified which kind of examination is meant

^g^ Briefly mentioned in the chapter "Why might a woman opt for oophorectomy over mastectomy?"

^h^Bilateral mastectomy for reduction of breast cancer risk

^i^Unclear as to whether unilateral or bilateral mastectomy is meant

^j^The term salpingo-oophorectomy is used in the DA glossary

^k^ The term oophorectomy is used in the DA tool

^l^States that oophorectomy reduces the risk of breast cancer in women at high risk of breast cancer

^m^Briefly mentioned on p. 9

^n^States that tamoxifen is most helpful for women under 50 years of age

^o^States on p. 2: "medications are being studied for breast cancer prevention"

^p^For post-menopausal women

^q^States that an international study examines the effect of anastrozole on preventing breast cancer in high-risk post-menopausal women

^r^Avoidance of hormone replacement therapy in post-menopause

^s^No increase in breast cancer risk if hormone replacement therrapy is stopped after menopause

^t^States that after surgically induced menopause younger women should consider short-term hormone replacement therapy up to the age of 50 to 52

^u^States that there is no evidence that screening leads to early diagnosis

^v^States that there is no evidence that screening saves lives

^w^Mentioned in the appendix

^x^States that in case of serious symptoms following removal of ovaries, women might consider to talk with the doctor about taking a short course of hormone therapy

^y^Not recommended/no clear statement as primary preventive measure in women with *BRCA1/2* mutations by the German S3 and S2 guidelines [8, 9, 14]

The analysis of the DAs in terms of the given target definitions is provided in Additional file 2.

The quality assessment revealed considerable differences between the seven DAs: one met all 16 of the IPDASi-SF criteria; one met 13 and five fulfilled seven to nine of them. In most cases information on the development and evaluation of the DA was lacking.

### Step 2: Determination of the medical content for the decision aids

The guidelines applicable in Germany are the S3 guidelines on (1) screening, diagnosis, therapy and aftercare for breast carcinomas [8]; (2) diagnosis, therapy and aftercare for malign ovarian tumours [14] and (3) of the Gynaecological Oncology Research Group (AGO) on diagnosis and therapy for early-stage and advanced breast carcinomas [9]. The versions valid at the time of DA development were used. The reference risk data for the general population was taken from the RKI [30, 31]. The data derived from the guidelines and the literature review indicated that previvors and survivors require different information due to the differences in risk data and health situations. Thus, two target-group-specific DA prototypes were designed.

### Step 3: Prototype development

The results of Steps 1 and 2 led to structure definitions for the form and content of the two DA prototypes, and to definition of the breakdown and the medical content required in each section. Both prototypes were generated as manuscripts with sketch illustrations. These were used as the basis for the focus group discussions and the internal clinical review process in Step 4.

### Step 4: Participation of target groups and internal clinical experts

In the focus group discussions with previvors (n = 9), the DA received a highly positive evaluation overall [65]. Participants considered it beneficial that all the information was presented in detail, compiled in one medium and met an adequate language and knowledge level. However, the group recommended that more psychological aspects be taken into account, the mutation be acknowledged as a stress factor, and certain aspects be repeated for emphasis. More information on the consequences of risk-reducing bilateral salpingo-oophorectomy and the procedures following the various breast surgeries was requested. Personal testimonies were also requested; yet, these were not included due to the lack of evidence for their benefits and their potential to cloud a person’s judgement [66].

The survivors (n = 10) also responded positively to the DA [67]. The volume and detail of the presented information were praised. Some participants also felt that certain sections should be more precise and comprehensible. More information was particularly requested on the following topics: BC on the affected side, BC treatment, risk of recurrence, and biological parameters. More detailed information was also requested on the procedures following risk-reducing breast surgery, breast reconstruction, symmetry following risk-reducing contralateral mastectomy, the consequences of risk-reducing bilateral salpingo-oophorectomy, and the intensified breast cancer screening and aftercare programme.

Both target groups assessed the integrated worksheets positively, but recommended replacing parts of the box-ticking sections with blank space to formulate and clarify their own thoughts and values. Both groups also expressed a wish for photos of genuine breast surgery results; these could not be provided for liability reasons. However, in the DAs the women are encouraged to seek advice from their surgeon about their individually planned surgery and ask for visual material of surgery results. In addition, they are encouraged to contact the self-help organisation BRCA-Netzwerk, which has many testimonials on this topic. For more details of the results of the focus group discussions with previvors and survivors, see Additional file 3.

The internal clinical expert reviews of the revised prototypes led to additional adjustments regarding language, updates, and explanations, particularly with regard to preventive options and their consequences. The results of the updated structure and content layout of the two DAs are listed in Table 4 for previvors and Table 5 for survivors.

**Table 4:** Structure and medical content of the DA for previvors with *BRCA1/2* mutations following incorporation of the results of the focus group discussions (DA A)

	Topic	Content
Information	Introduction	Addressing the target group and target definition Notes on authors, funding source and use of the decision aid
	(1) Overview	Overview of the contents of this decision aid
	(2) What does a mutation in the *BRCA1* or *BRCA2* gene mean?	Function of the non-mutated *BRCA* genes Significance of hereditary *BRCA* mutations Average risks of breast cancer and ovarian cancer each subdivided into *BRCA1* and *BRCA2* mutations Personal risk of breast cancer and ovarian cancer
	(3) What consequences can I expect based on the result of my genetic test?	Overview of potential preventive options Intensified breast cancer screening programme: Aims, reliability, procedure, implementation, pros/cons, overview table Risk-reducing bilateral mastectomy: Effect on risk of developing breast cancer Forms of mastectomy, pros/cons, overview table Forms of breast reconstruction, pros/cons, overview table Risk-reducing removal of both ovaries and Fallopian tubes: Effect on risk of developing ovarian cancer and survival, surgical procedure, option for hormone replacement therapy, pros/cons, overview table FAQs on other aspects
Support of decision-making	(4) How do I work out my own perspective? A guide to making an informed decision	Worksheet 1: Comparison of preventive options Worksheet 2: Significance of certain aspects in terms of your own cancer risk (clarifying values and preferences; box-ticking) Worksheet 3: A step-by-step guide to making your decision (your situation, clarifying values and preferences; free-form notes) Worksheet 4: Helping you prepare for your consultation with your doctor
	(5) Other helpful information	Points of contact for personal advice, self-help What can I do for myself? Tips for additional information material Glossary Contact addresses and links References
	(6) Appendix	Fact boxes for women with *BRCA1* and *BRCA2* mutations: Average risks of developing breast cancer: Lifetime, age and time-related (10 year) risks Average risks of developing ovarian cancer: Lifetime, age and time-related (10-year) risks

*BRCA1* BReast CAncer gene 1, *BRCA2* BReast CAncer gene 2, *DA* decision aid, *FAQs* frequently asked questions

**Table 5:** Structure and medical content of the DA for survivors with *BRCA1/2* mutations following incorporation of the results of the focus group discussions (DA B)

	Topic	Content
Information	Introduction	Addressing the target group and target definition Notes on authors, funding source and use of the decision aid
	(1) Overview	Overview of the contents of this decision aid
	(2) What does a mutation in the *BRCA1* or *BRCA2* gene mean?	Function of the non-mutated *BRCA* genes Significance of hereditary *BRCA* mutations Risks of developing contralateral breast cancer and ovarian cancer (lifetime, age- and time-related risks) each subdivided into *BRCA1* and *BRCA2* mutations Personal risk of breast cancer (opposite side, affected side) Competing risks
	(3) Understanding your diagnosis and treatment of breast cancer	Information with regard to the affected breast Stages, types and spread of breast cancer Potential characteristics of breast cancer for women with *BRCA1* and *BRCA2* mutations Basic treatment steps: breast surgery, radiotherapy, medication Aftercare
	(4) What consequences can I expect based on the result of my genetic test?	Overview of potential preventive options Intensified breast cancer screening and aftercare programme: Aims, reliability, procedure, implementation, pros/cons, overview table Risk-reducing contralateral mastectomy (of the healthy opposite side): Effect on risk of developing breast cancer and survival Forms of mastectomy, pros/cons, overview table Forms of breast reconstruction, pros/cons, overview table Risk-reducing removal of both ovaries and Fallopian tubes: Effect on risk of developing ovarian cancer and survival, surgical procedure, options for relief of menopausal symptoms, pros/cons with overview table FAQs on other aspects
Support of decision-making	(5) How do I work out my own perspective? A guide to making an informed decision	Worksheet 1: Comparison of preventive options Worksheet 2: Significance of certain aspects in terms of your own cancer risk (clarifying values and preferences; box-ticking) Worksheet 3: A step-by-step guide to making your decision (your situation, clarifying values and preferences; free-form notes) Worksheet 4: Helping you prepare for your consultation with your doctor
	(6) Other helpful information	Points of contact for personal advice, self-help What can I do for myself? Tips for additional information material Glossary Contact addresses and links References
	(7) Appendix	Fact boxes for women with *BRCA1-* and *BRCA2* mutations: Average risks of developing contralateral breast cancer: Lifetime, age and time-related risks Average risks of developing ovarian cancer: Lifetime, age and time-related risks

*BRCA1* BReast CAncer gene 1, *BRCA2* BReast CAncer gene 2, *DA* decision aid, *FAQs* frequently asked questions

### Step 5: Validation by external experts

The external expert validation process led to revisions and expansion of the topics, particularly relating to risk-reducing breast surgery, including practical information such as surgery time, length of stay in hospital, and need for follow-up surgery. The self-help experts’ reviews revealed a need for more information and assistance, e.g. on dealing with the mutation, preventive options and their consequences, the decision-making process, and practical information, e.g. for women who decide against reconstruction.

### Step 6: User tests

The DAs for previvors (n = 6) and survivors (n = 5) were both rated positively, in particular in terms of length, balanced presentation of options, usefulness for decision-making, sufficient information to make decisions, satisfaction, and likelihood of recommendation to others. Minor content adjustments were required. For more details on the results of the user tests including the underlying interview guideline, see Additional file 4.

## Discussion

In this study, two structured, evidence-based DAs were developed for previvors and survivors with *BRCA1/2* mutations to support their decision-making on risk-adapted preventive options for BC and OC. The DAs were developed by a multidisciplinary team that included experts with extensive experience in specialised counselling for the target groups. The six-stage development process was based on the IPDAS criteria. It included literature reviews of available DAs and current medical evidence, internal validation by clinical experts, participation from external previvors/survivors, and validation by external medical specialists and self-help experts. None of the external persons were involved in the development process.

With its multi-level validation and the involvement of independent members of the target groups and external experts, this comprehensive development process is a high-quality procedure based on established approaches [35, 40, 46]. In a deviation from the IPDAS requirements, no separate needs analysis was conducted for the target doctors, as the specialists’ needs were taken into account and reflected throughout the development process due to the direct involvement of specialised medical consultants in the development team.

In principle, the development of DAs should be seen as an innovative approach in Germany, where only a modest number of DAs have been created and are in use [68, 69]. Experience in this country is therefore limited. There are several DAs for women with *BRCA1/2* mutations on the international stage [29]. However, the literature review within the presented development process showed that these would need to be adapted to suit the German healthcare setting and sociocultural and socio-economic parameters. The finding that available international DAs for women with *BRCA1/2* mutations partly deviate considerably from the recommendations of the German guidelines and are therefore not transferable to the German context could be verified by a recently updated and widely expanded literature review on this topic [70].

During the development process, it became clear that the medical information required by the two target groups differed in some ways, and that they seemed to require different levels of information on certain aspects. For instance, the risks of first BC and contralateral BC [1, 71], and the respective surgery options have different effects. Furthermore, mutation carriers with prior BC face different considerations than those without a history of cancer. Information needs may also differ in the two target groups [2]. This was supported by the focus group discussions. Survivors requested a lot of information on BC on the affected side, its treatment and the risk of recurrence, while previvors were more interested in information on risk-reducing surgeries and their consequences.

These DAs provide differentiated risk information for women with *BRCA1* and *BRCA2* mutations and address questions relating to dealing with both the BC risk and the OC risk. This represents an advantage compared to international DAs, which do not differentiate between *BRCA1* and *BRCA2* mutation carriers with and without a history of BC [72, 73], which address a broader target group of women with an increased risk of BC and/or OC [59–61, 64, 74, 75], and which primarily tackle either the risks of BC [59, 64] or OC [60, 63, 74–76]. There are very few DAs specifically designed for women with *BRCA1/2* mutations with a history of BC [77]. This could be due to the complexity of the information regarding decisional options, the personal situation of these women, and the number of individual factors involved.

This project resulted in two structured, evidence-based DAs for women with *BRCA1/2* mutations, each of which is aimed at a clearly defined target group (previvors/survivors) and the content of which is tailored to the respective needs of each target group. After evaluation of their effectiveness and acceptability in clinical use in a randomised controlled trial, both DAs will be available as printed paper brochures to be used in post-test genetic counselling and given to women to take home. Both DAs will also be available as electronic versions that can be downloaded in PDF format. A full revision and update is scheduled for 2 years after completion of the final versions of the DAs.

The strengths of this study include the systematic DA development based on IPDAS criteria and evidence-based medicine following clearly defined and sequential development steps. These ensured that the development and its documentation remained transparent and the DAs developed meet high-quality standards. The literature reviews in Steps 1 and 2 provided a broad basis for defining the basic structural elements, the content structure and the contents of the DAs. For the actual creation of the two DAs, starting with step 3, independent target group persons were included for each development step in order to discuss and evaluate the respective versions from their perspective and thus support patient orientation. It has proven effective and is increasingly recommended to involve the target groups for decision-making-support and shared decision-making tools in the development process [40, 46, 68, 78]. A conscious effort was made to involve expert patients who play an active role in self-help and have an insight into the different perspectives of women with* BRCA1/2* mutations, as well as layperson-patients with no active role, who contribute their very own perspective. Involving this range of women with* BRCA1/2* mutations could increase the acceptance, relevance and practical applicability of the DAs in daily clinical work. Another strength is the way the DAs clearly address and are aimed at specific target groups, and the level of detail they provide. Each DA version offers its target group the information they need and want on the context, risks, preventive options and questions to consider. Both DAs also provide detailed responses to questions regarding preventive measures for BC and OC.

As the systematic literature review of existing DAs in Step 1 was conducted at the start of the project, there is a limit to how up-to-date the identified DAs may be. However, the basic findings of this review have recently been confirmed [70]. Another limitation is the lack of a systematic evidence review in all parts of the development process in Step 2. On the other hand, it makes sense to use the evidence-based S3 and S2 guidelines that apply to the German healthcare setting as a basis for developing German DAs and include further evidence-based content to make up for missing information. Another limitation is a selection bias resulting from the purposive selection of volunteer target group participants for the focus groups, the external reviews by expert patients and the user tests. Any distortions that may occur due to the expert patients’ advanced knowledge [79] were counteracted by also involving layperson-patients. A limitation that may arise from the restricted number of target group persons in the final user tests may be mitigated by the fact that both DAs will be tested in an evaluation study. The evaluation for effectiveness and acceptability in clinical use is part of the final quality assurance [35, 40] and a randomised controlled study of both DAs is currently under way (DRKS00015823).

## Conclusions

A comprehensive work process based on high-quality standards was used to develop the first evidence-based, structured DAs for previvors and survivors with *BRCA1/2* mutations for Germany. They are designed to support these women in coming to an informed, high-quality decision on what preventive measures they wish to take at what time, taking into account their own values and preferences. As patient-oriented tools, these DAs represent an innovative addition to the range of specialised consulting services offered by the GC-HBOC’s 24 centres and the affiliated breast centres. Their implementation in specialised care will be an important step in increasing the autonomy of women with *BRCA1/2* mutations.

## Supplementary Information

**Additional file 1.** Method details Step 1–6. Table: Development of decision aids for women with BRCA1 and BRCA2 mutations - Methodological approach Steps 1–6.

**Additional file 2.** Identified decision aids. **Table S1** Systematic literature research: List of identified decision aids. **Table S2** Systematic literature research: Evaluation of the identified decision aids with regard to the given target definitions.

**Additional file 3.** Focus group discussions. **Table S1** Characteristics of the focus group participants. **Table S2** Basic results of the focus group discussions with previvors and survivors.

**Additional file 4.** User tests. **Table S1** Basic results of the user tests with previvors (n = 6) for DA A. **Table S2** Basic results of the user tests with survivors (n = 5) for DA B. **Table S3** Interview guideline for the user test of the beta version of decision aid A for previvors and decision aid B for survivors..

## Data Availability

The datasets generated during and/or analysed during the current study are available from the corresponding author on reasonable request.

## Abbreviations

AGO: Arbeitsgemeinschaft Gynäkologische Onkologie (Working Group for Gynaecological Oncology)
BC: breast cancer
*BRCA1/2*: BReast CAncer gene 1 and/or 2
DAs: decision aids
GC-HBOC: German Consortium of Hereditary Breast and Ovarian Cancer
IPDAS: International Patient Decision Aid Standards
IPDASi-SF: IPDAS instrument short form
LICS: lobular in situ carcinoma
MRI: magnetic resonance imaging
OC: ovarian cancer
OHRI: Ottawa Hospital Research Institute
PDF: portable data format
RKI: Robert Koch Institute

## Footnotes

1 BRCA-Netzwerk: German self-help organisation (registered association) to support persons with familial cancers, in particular familial breast and ovarian cancer, e.g. due to *BRCA1/2* mutations (high-risk or affected persons).
