# Supplementary material for: Development of decision aids for female BRCA1 and BRCA2 mutation carriers in Germany to support preference-sensitive decision-making
Source: BMC Med Inform Decis Mak. 2021 Jun 5;21:180. doi: 10.1186/s12911-021-01528-4 (PMC8180100; doi:10.1186/s12911-021-01528-4)
Supplement: Supplementary file 2 — Additional file 2. Identified decision aids. Table S1 Systematic literature research: List of identified decision aids. Table S2 Systematic literature research: Evaluation of the identified decision aids with regard to the given target definitions. [file 12911_2021_1528_MOESM2_ESM.pdf]

Table 1: Systematic literature research: List of identified decision aids

Authors/Developers [Reference]	Title of the decision aid	Year of publication	Country	Target group addressed	Cancer risk addressed	Basic structural elements		Format
Metcalfe K, Narod S, Poll A, O'Connor A.<br>The Centre for Research in Women's Health, Toronto.<br>University of Ottawa.<br>[57]	What are my options for Breast Cancer Prevention?<br>Facts and Decision Aid for Women with a *BRCA1* or *BRCA2* mutation.	2006	Canada	Women with *BRCA1/2* mutations;<br>• No differentiation between *BRCA1* and *BRCA2* mutation.<br>• Addresses not affected by cancer (previvors)	Breast cancer risk	Target group, target decision		Brochure / PDF
						Medical information	• Risks due to *BRCA1/2* mutation<br>• Average morbidity rates for BC/OC<br>• Preventive options for BC<br>• Outcomes and consequences of preventive options<br>• Benefits and risks of preventive options<br>• Fact boxes	
						Tools for decision-making support	• General information (short)<br>• Step-by-step guide<br>• Clarifying values/preferences	
						Other	• Glossary<br>• Reference list / further literature	
Kurian AW, Plevetris S.<br>Stanford University School of Medicine.<br>[58]	Decision tool for women with *BRCA* Mutations.	2011	USA	Women with *BRCA1/2* mutations;<br>• Clear differentiation between *BRCA1* and *BRCA2* mutation.<br>• Addresses not affected by cancer (previvors).	Breast cancer risk,<br>Ovarian cancer risk	Target group, target decision		Online tool for individu-alisation
						Medical information	• Risks due to *BRCA1/2* mutation<br>• Average morbidity rates for BC/OC<br>• Preventive options<br>• Outcomes and consequences of preventive options	
						Other	• Glossary<br>• Contact addresses and/or internet links<br>• Reference list/further literature	
Hereditary Cancer Clinic, Prince of Wales Hospital, Centre for Genetics Education, NSW Health, Royal North Shore Hospital.<br>[64]	Information for women considering preventive mastectomy because of a strong family history of breast cancer.	2012	Australia, New South Wales	Women with a strong family history of breast cancer and<br>• *BRCA1/2* mutations<br>• Personal history of unilateral breast cancer<br>• Personal history of multiple breast biopsies<br>• Breast cancer anxiety	Breast cancer risk	Target group, target decision		Brochure / PDF
						Medical information	• Family history of BC<br>• Risks due to *BRCA1/2* mutation<br>• Example age-/time-related risk of BC<br>• Preventive options<br>• Outcomes and consequences of preventive options<br>• Benefits and risks of preventive options<br>• Personal stories	
						Tools for decision-making support	• General information (detailed)<br>• Question list addressing medical information/docter patient dialogue	
						Other	• Contact addresses and/or internet links<br>• Reference list/further literature	

Authors/Developers [Reference]	Title of the decision aid	Year of publication	Country	Target group addressed	Cancer risk addressed	Basic structural elements		Format
Healthwise Cancer Clinic.<br>[61]	Breast Cancer: What Should I Do if I'm at High Risk?	2016*	USA	Women at high risk for breast cancer with<br>• Family history of breast cancer<br>• *BRCA1/2* mutations	Breast cancer risk	Target group, target decision		Online text
						Medical information	• Family history of BC<br>• Risks due to *BRCA1/2* mutation<br>• Average morbidity rates for BC<br>• Preventive options<br>• Outcomes and consequences of preventive options<br>• Benefits and risks of preventive options<br>• Personal stories<br>• Fact boxes	
						Tools for decision-making support	• Clarifying values/preferences	
						Other	• Reference list / further literature	
Mayo Clinic Staff.<br>[59]	Preventive (prophylactic) mastectomy: Surgery to reduce breast cancer risk.	2016*	USA	Women at increased risk for breast cancer with<br>• Personal history of unilateral breast cancer<br>• Family history of breast cancer<br>• *BRCA1/2* mutations<br>• Personal history of LCIS<br>• Personal history of radiotherapy at the age of 10 to 30<br>• Dense breast tissue	Breast cancer risk	Target group, target decision		Online text on website
						Medical information	• Family history of BC<br>• Preventive options<br>• Benefits and risks of preventive options	
						Other	• Reference list / further literature	
Cardiff University.<br>[62, 63]	OVDex. Oophorectomy - Decision Explorer.	2014	UK, Wales	Women at increased risk of ovarian cancer with<br>• *BRCA1* or *BRCA2* mutations<br>• Being from a Lynch syndrome family<br>• No genetic test<br>• Uninformative genetic test<br>• Negative genetic test	Ovarian cancer risk	Target group, target decision		Online tool for individuali sation
						Medical information	• Family history of OC<br>• Risks due to *BRCA1/2* mutation<br>• Average morbidity rates for OC<br>• Preventive options<br>• Outcomes and consequences of preventive options<br>• Benefits and risks of preventive options<br>• Hormone replacement therapy to counteract potential undesired effects of risk-reducing bilateral salpingo-oophorectomy	
						Tools for decision-making support	• Step-by-step guide<br>• Clarifying values/preferences	
						Other	• Contact addresses and/or internet links<br>• Reference list / further literature	

<table>
<tr><th>Authors/Developers
[Reference]</th><th>Title of
the decision aid</th><th>Year of publication</th><th>Country</th><th>Target group addressed</th><th>Cancer risk addressed</th><th colspan="2">Basic structural elements</th><th>Format</th></tr>
<tr><td rowspan="4">Mayo Clinic Staff.
[60]</td><td rowspan="4">Prophylactic oophorectomy: Preventing cancer by surgically removing your ovaries</td><td rowspan="4">2014*</td><td rowspan="4">USA</td><td rowspan="4">Women at increased risk of ovarian cancer with
• BRCA1 or BRCA2 mutations
• Strong family history of breast cancer and ovarian cancer without known genetic alteration
• Strong likelihood of gene mutation, but no genetic testing</td><td rowspan="4">Ovarian cancer risk</td><td colspan="2">Target group, target decision</td><td rowspan="4">Online text</td></tr>
<tr><td>Medical information</td><td>• Family history of OC
• Risks due to BRCA1/2 mutation
• Preventive options
• Outcomes and consequences of preventive options
• Benefits and risks of preventive options
• Hormone replacement therapy to counteract potential undesired effects of risk-reducing bilateral salpingo-oophorectomy</td></tr>
<tr><td>Tools for decision-making support</td><td>• Question list addressing medical information/docter patient dialogue</td></tr>
<tr><td>Other</td><td>• Reference list / further literature</td></tr>
</table>

** a current version dated 2019 is available*

Table 2: Systematic literature research: Evaluation of the identified decision aids with regard to the given target definitions

		Requirements of target definitions met?						
Target	Target definition	Metcalfe, 2006 [57]	Kurian, 2011 [58]	Hereditary Cancer Clinic et al, 2012 [64]	Healthwise, 2016 [61]	Mayo Clinic, 2016 [59]	Cardiff University, 2014 [62, 63]	Mayo Clinic, 2014 [60]
Target group	The DA should be designed for female previvors and survivors with pathogenic *BRCA1* or *BRCA2* mutations.	partly met	partly met	partly met	partly met	partly met	partly met	partly met
Target decision	The addressed decision situation should be the choice of an individual preventive strategy.	met	met	met	met	met	met	met
Decision-making-related target	The DA should help previvors and survivors make informed decisions and clarify their individual values and preferences.	partly met	partly met[1]	partly met	partly met	partly met[1]	partly met	partly met
Information/ content-related target	The DA should address the risks of breast cancer and ovarian cancer and present the respective preventive options, including their consequences. Existing data on lifetime and on age and time-related risks should be taken into account in order to facilitate the women's risk perception within a foreseeable period of time.	partly met	partly met	partly met	partly met	partly met	partly met	partly met
Needs-related target	The DA should fulfil the specific needs of the target group.	met	partly met	partly met	partly met	partly met	partly met	partly met
Evidence-related target	The medical content should be based on the currently applicable German S3 and S2 guidelines[2] and, where necessary, on additional data with a high level of evidence; [preferably data with an expected level of evidence of IIb and higher (e.g. systematic reviews based on RCTs, meta-analyses, RCTs, high quality cohort studies)]	not met[3]	not met[3]	not met[3]	not met[3]	not met[3]	not met[3]	not met[3]

DA: decision aid; [1]DA does not contain any tool for supporting decision making;[2]the German S3 guidelines represent the evidence-based consensus in Germany at the highest quality level of methodological development; [3]none of the DAs used the German S3 guidelines as a basis.
