# Supplementary material for: Development of decision aids for female BRCA1 and BRCA2 mutation carriers in Germany to support preference-sensitive decision-making
Source: BMC Med Inform Decis Mak. 2021 Jun 5;21:180. doi: 10.1186/s12911-021-01528-4 (PMC8180100; doi:10.1186/s12911-021-01528-4)
Supplement: Supplementary file 3 — Additional file 3. Focus group discussions. Table S1 Characteristics of the focus group participants. Table S2 Basic results of the focus group discussions with previvors and survivors. [file 12911_2021_1528_MOESM3_ESM.pdf]

Table 1: Characteristics of the focus group participants

Criterion	Target group<br>Previvors	 Survivors
**Total number (n)**	9	10
**Medium age [range] (years)**	41,1 [27-53]	45,6 [27-56]
**Gene test result**		
BRCA1 mutation (n)	6	8
BRCA2 mutation (n)	3	1
BRCA1 and BRCA2 mutation (n)	0	1
Genetic test result received [range] (months ago)	7,5 [4-15]	30 [4-156]
**History of breast cancer**		
Individuals with one breast cancer (n)	not applicable	5
Individuals with more than one breast cancer (n)	not applicable	5
Therapeutic surgery: breast-conserving (n of breasts)	not applicable	10
Therapeutic surgery: mastectomy (n of breasts)	not applicable	4
Therapeutic surgery: not specified (n of breasts)	not applicable	1
**Preventive measures taken - breast cancer**		
Intensified breast cancer screening (n)	3	3
Risk-reducing mastectomy (n)	4	4
- With breast reconstruction (n)	4	4
- Without breast reconstruction (n)	0	0
**Preventive measures planned - breast cancer**		
Intensified breast cancer screening (n)	2	1
Risk-reducing mastectomy definitely planned (n)	0	0
Considering risk-reducing mastectomy (n)	3	1
Definitely no risk-reducing mastectomy (n)	2	0
**Preventive measures taken - ovarian cancer**		
Risk-reducing bilateral salpingo-oophorectomy (n)	4	6
**Preventive measures planned - ovarian cancer**		
Risk-reducing bilateral salpingo-oophorectomy definitely planned (n)	3	2
Considering risk-reducing salpingo-oophorectomy (n)	0	1
**Other**		
Currently no further planning (n)	1	1
Planning not specified / not applicable (n)	1	3
**Family history of breast and/or ovarian cancer**		
Yes (n)	9	4
No (n)	0	3
Not specified / not applicable (n)	0	3
**Family planning**		
Completed / has own children (n)	7	not specified
Not completed / has childbearing preferences (n)	2	not specified

Table 2: Basic results of the focus group discussions with previvors and survivors

Focus group discussions	Topic	Basic results
**With previvors (n=9)**	General impression	• Highly positive to the DA overall.<br>• High, but suitable level, most of the information is well explained and comprehensible.
	Beneficial aspects	• Information in detail compiled in one medium, of a suitable level; makes it possible to delve deeper into certain topics.<br>• Detailed information especially on mutations, risks, risk-reducing breast surgery.<br>• Integrated overview tables and fact boxes.<br>• Worksheets for comparing the options and establishing values.<br>• Integrated glossary.
	Aspects to add, delete, or change	• To clarify that the mutation should be acknowledged a stress factor, even though not having cancer.<br>• To address positive and negative consequences of the options.<br>• To change the sections for clarifying values in the worksheets from box ticking to free-form notes to formulate own thoughts.<br>• To present personal testimonies.[1]<br>• To integrate photos of genuine breast surgery results.[2]
	Specific needs	• More information on the consequences of risk-reducing salpingo-oophorectomy (e.g. loss of fertility, hormone replacement therapy, adverse effects).<br>• More information on the various procedures for risk-reducing breast surgeries and the procedure following the operation (e.g. stay in the clinic, duration of recovery).<br>• More psychological aspects.
**With survivers (n=10)**	General impression	• Positive response to the DA overall.
	Beneficial aspects	• Information in detail compiled in one medium; makes it possible to delve deeper into certain topics.<br>• Comprehensive information, especially on breast surgery and reconstruction.<br>• Worksheets for comparing the options and establishing values.
	Aspects to add, delete, or change	• To make certain sections more precise and comprehensible.<br>• To handle breast cancer self-examination as an important topic.<br>• To change the sections for clarifying values in the worksheets from box ticking to free-form notes to formulate own thoughts.<br>• To integrate summary boxes at the end of each chapter.<br>• To integrate photos of genuine breast surgery results.[2]
	Specific needs	• More information on the first breast cancer on the already affected side: biochemical parameters, therapy of the first breast cancer including surgery, radiotherapy, medical therapy (anti-hormonal, anti-growth receptor, chemotherapy), risk of recurrence on the affected side.<br>• More information on the procedure following the various breast surgeries (e.g. stay in the clinic, duration of recovery), breast reconstruction, and symmetry following contralateral mastectomy of the opposite side.<br>• More information on the consequences of risk-reducing salpingo-oophorectomy.<br>• More information on the intensified breast screening and aftercare programme.

[1]Personal testimonies were not included due to the lack of evidence for their benefits and their potential to cloud a person's judgement [66]. [2]Photos of genuine breast surgery results were not provided for liability reasons.
