# Supplementary material for: Development of decision aids for female BRCA1 and BRCA2 mutation carriers in Germany to support preference-sensitive decision-making
Source: BMC Med Inform Decis Mak. 2021 Jun 5;21:180. doi: 10.1186/s12911-021-01528-4 (PMC8180100; doi:10.1186/s12911-021-01528-4)
Supplement: Supplementary file 4 — Additional file 4. User tests. Table S1 Basic results of the user tests with previvors (n = 6) for DA A. Table S2 Basic results of the user tests with survivors (n = 5) for DA B. Table S3 Interview guideline for the user test of the beta version of decision aid A for previvors and decision aid B for survivors.. [file 12911_2021_1528_MOESM4_ESM.pdf]

Table 1: Basic results of the user tests with previvors (n=6) for DA A

(a) DA A Acceptability criteria	Assessment	(n)
General impression, spontaneous assessment	Positive	6
	Partly positive/Partly negative	0
	Negative	0
Scope of the DA (length)	Just right	6
	Too long	0
	Too short	0
Amount of information	Just right	4
	Too much	0
	Partly too little	2
	Too little	0
Balanced presentation of the options	Well balanced	6
	Unbalanced	0
Comprehensibility	Easy to understand	3
	Partly good /partly difficult to understand	3
	Difficult to understand	0
Usefulness in terms of decision making	High	6
	Moderate	0
	Low	0
Satisfaction with the DA	High	6
	Moderate	0
	Low	0
Sufficient information for decision making	Yes	6
	No	0
	I don't know	0
Would you recommend the DA to other women in your situation?	Yes	6
	No	0
	I don't know	0

(b)	DA A Content	Adjustments after user testing
	Introduction	Minor adjustments: additional information
1.	Overview	No adjustments
2.1	Information on mutations in the genes *BRCA1* or *BRCA2*	Minor adjustments: Revision of some of the explanatory texts
2.2	Information on the risks of developing breast and ovarian cancer	No adjustments
3.1	Preventive option: Intensified breast cancer screening	Adjustments: Revision of two figures with legends
3.2	Preventive option: Risk-reducing bilateral mastectomy	Minor adjustments: additional information
	Forms of breast reconstruction	Minor adjustments: Revision of some of the explanatory texts
3.3	Preventive option: Risk-reducing removal of both ovaries and Fallopian tubes	No adjustments
4.	Worksheets 1 to 4 for support of decision-making	Minor adjustments in Worksheets 2, 3 and 4
5.	Further information	Minor adjustments: additional information
6.	Appendix: Fact boxes	No adjustments

Table 2: Basic results of the user tests with survivors (n=5) for DA B

(a) DA B Acceptability criteria	Assessment	(n)
General impression, spontaneous assessment	Positive	4
	Partly positive/Partly negative	1
	Negative	0
Scope of the DA (length)	Just right	4
	Too much	(1*)
	Too short	0
	Not specified**	1
Amount of information	Just right	3
	Too much	2
	Too little	0
Balanced presentation of the options for the healthy breast (opposite side)	Well balanced	5
	Unbalanced	0
Balanced presentation of the option for both ovaries and Fallopian tubes	Well balanced	3
	Not well balanced	1
	I don't know	0
	Not specified**	1
Comprehensibility	Easy to understand	4
	Partly good /partly difficult to understand	1
	Difficult to understand	0
Usefulness in terms of decision making	High	5
	Moderate	0
	Low	0
Satisfaction with the DA	High	4
	Moderate	1
	Low	0
Sufficient information for decision making	Yes	4
	No	0
	I don't know	0
	Not specified	1
Would you recommend the DA to other women in your situation?	Yes	5
	No	0
	I don't know	0

**additional comment from n=1 on assessment for women receiving chemotherapy **one participant did not answer*

(b)	DA B Content	Adjustments after user test
	Introduction	Minor adjustments: language revision
2.	Overview	No adjustments
2.2	Information on mutations in the genes *BRCA1* or *BRCA2*	Adjustment: Addition of a summary
2.2	Information on the risks of developing breast cancer in the healthy breast (opposite side) and of developing ovarian cancer;<br>Information on the risk of recurrence on the affected side	No adjustments
3.	Information on diagnosis and treatment of breast cancer (already affected side)	Adjustments: Addition of a summary
4.1	Preventive option: Intensified breast cancer screening and aftercare of the breast	Minor adjustments: language revision
4.2	Preventive option: Risk-reducing removal of the healthy breast (opposite side)	Minor adjustments: text completion
	Forms of breast reconstruction	No adjustments
4.3	Preventive option: Risk-reducing removal of both ovaries and Fallopian tubes	Minor adjustments: text completion
5.	Worksheets 1 to 4 for support of decision-making	Minor adjustments: Worksheet 2
6.	Further information	No adjustments
7.	Appendix: fact boxes	No adjustments

Table 3: Interview guideline for the user test of beta version of decision aid A for previvors and decision aid B for survivors.

(Part 1) General impression / acceptability (closed questions)

First, I would like to know what your general impression is of the decision aid. I will now ask you some questions. Please answer questions using the answers provided. However, please feel also free to comment on each point individually.

**1**	First, I would like to hear from you spontaneously what your general impression of the decision aid is. What would you like to tell me spontaneously? (free answer)<br>How would you rate your general impression?<br>☐ Positive impression<br>☐ Partly positive / Partly negative impression<br>☐ Negative impression
**2**	Now come some specific questions. How do you rate the scope of the decision aid?
	The decision aid<br>☐ Is too long<br>☐ Is too short<br>☐ Has exactly the right length
**3**	How do you rate the amount of information?
	The decision aid contains<br>☐ Too much information<br>☐ Too little information<br>☐ Exactly the right amount of information
**4**	How balanced are the options for action "intensified breast cancer screening" and "risk-reducing surgeries"?
	The options for action are<br>☐ Presented in a balanced way<br>☐ Unbalanced, in favour of intensified breast cancer screening<br>☐ Unbalanced, in favour of risk-reducing surgeries
**5**	How comprehensible is the information?
	The information in this decision aid is<br>☐ Easy to understand<br>☐ Partly good, partly difficult to understand<br>☐ Difficult to understand
**6**	How would you rate the benefit of the decision aid to make a definite decision for a preventive option?
	I rate the benefit as<br>☐ high<br>☐ moderate<br>☐ low

7 How satisfied are you with this decision aid?

- [ ] Very satisfied/satisfied
- [ ] Moderately satisfied
- [ ] Dissatisfied/very dissatisfied

8 Do you think that the decision aid provides enough information to enable women with *BRCA1/2* mutations to find a well-suited decision for a preventive option?

- [ ] Yes
- [ ] No
- [ ] I don't know

9 Would you recommend this decision aid to other women in your situation?

- [ ] Yes
- [ ] No
- [ ] I don't know

Questionnaire of Part 1 adapted from: [55] O'Connor AM, Cranney A. User Manual - Acceptability. 1996, updated 2002. Ottawa Hospital Research Institute, Ottawa, Canada. //decisionaid.ohri.ca/docs/develop/User_Manuals/UM_Acceptability.pdf. Accessed 7 March 2017; and [56] Metcalfe KA, Poll A, O'Connor A, Gershman S, Armel S, Finch A, Demsky R, Rosen B, Narod SA. Development and testing of a decision aid for breast cancer prevention for women with a BRCA1 or BRCA2 mutation. Clin Genet. 2007;72:208-17.

Note: The main aim is for each test reader to completely answer the questions in part 1 on acceptability. The aspects of parts 2 to 4 should also be covered as completely as possible, but only to the extent that each test reader is willing to comment on them in each case.

(Part 2 for DA A) Impressions on the contents of the decision aid A (free comments)

Now I would like to hear your opinion on certain topics in this decision aid. Please feel free to comment on any topics that you would like to share with us.

**1**	Chapter 1:	Overview
**2**	Chapter 2.1:	Information on mutations in the genes *BRCA1* and *BRCA2*
**3**	Chapter 2.2:	Information on the risks of breast and ovarian cancer.
**4**	Chapter 3.1:	Preventive option: Intensified breast cancer screening
**5**	Chapter 3.2:	Preventive option: Risk-reducing bilateral mastectomy<br>Forms of breast reconstruction
**6**	Chapter 3.3:	Preventive option: Risk-reducing removal of both ovaries and Fallopian tubes
**7**	Chapter 3.4:	Frequent Questions and answers
**8**	Chapter 5:	Further information
**9**	Appendix:	Fact boxes

(Part 2 for DA B) Impressions on the contents of the decision aid B (free comments)

Now I would like to hear your opinion on certain topics in this decision aid. Please feel free to comment on any topics that you would like to share with us.

**1**	Chapter 1:	Overview
**2**	Chapter 2.1:	Information on mutations in the genes *BRCA1* and *BRCA2*
**3**	Chapter 2.2:	Information on the risks of contralateral breast cancer in the healthy breast (opposite side) and the risks of ovarian cancer.
**4**	Chapter 2.3:	Information on the individual risk of breast cancer recurrence in the affected side
**5**	Chapter 3:	Information on diagnosis and treatment of breast cancer (already affected side)
**6**	Chapter 4.1:	Preventive option: Intensified breast cancer screening and aftercare of the breast
**7**	Chapter 4.2:	Preventive option: Risk-reducing contralateral mastectomy<br>Forms of breast reconstruction
**8**	Chapter 4.3:	Preventive option: Risk-reducing removal of both ovaries and Fallopian tubes
**9**	Chapter 4.4:	Frequent Questions and answers
**10**	Chapter 5:	Further information
**11**	Appendix:	Fact boxes

(Part 3) Questions on the work sheets 1 to 4 or free comments (closed questions or free comments)

In the following, I am interested on how you would rate worksheets 1 to 4 in chapter 4. If you wish, please answer the following questions, but you are also welcome to answer freely.

1 How would you rate worksheet 1:
"Comparison of the different prevention measures for women with *BRCA1/2* mutation"

Work sheet 1 is

- ☐ useful
- ☐ not useful

2 How would you rate worksheet 2:
"What aspects are important to me in terms of my cancer risk?"

Work sheet 2 provides

- ☐ Good support to get clarity about how to deal with the risks of cancer
- ☐ No support in getting clarity about how to deal with the risks of cancer

3 How would you rate worksheet 3:
"Step by step to the decision - creating free notes"

Work sheet 3

- ☐ Helps to make a decision
- ☐ Makes it difficult to make a decision
- ☐ Does not help to make a decision

4 How would you rate work sheet 4:
“Aids to prepare for the doctor’s consultation”

Work sheet

- ☐ Helps well in preparing for the doctor-patient interview.
- ☐ Does not help to prepare for the doctor-patient interview.

(Part 4) What else is important to you? (free comments)

Do you have any more comments on the decision aid that you would like to share with us?
Criticisms? Praise? Suggestions?
